# Valorization of Hydrogen Peroxide for Sodium Percarbonate and Hydrogen Coproduction via Alkaline Water Electrolysis: Conceptual Process Design and Techno-Economic Evaluation

**DOI:** 10.1021/acs.iecr.4c03408

**Published:** 2025-01-24

**Authors:** Mahdi Mohajeri, Shachi Shanbhag, Eleftherios Trasias, Farzad Mousazadeh, Wiebren de Jong, Sohan A. Phadke

**Affiliations:** † Chemical Engineering Department, Delft University of Technology, Delft 2629 HZ, The Netherlands; ‡ Process & Energy, Mechanical Engineering, Delft University of Technology, Delft 2628 CB, The Netherlands

## Abstract

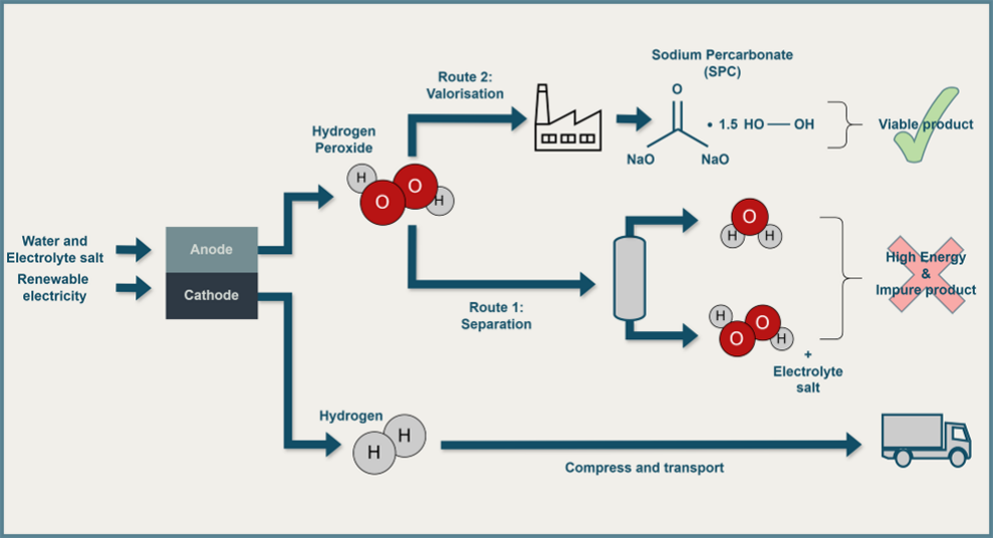

The recent interest in the production of green hydrogen through water electrolysis is hampered by its high cost when compared to steam methane reforming. To overcome this disadvantage, some studies explore replacing oxygen production with hydrogen peroxide at the anode, which has a higher value. Existing electrocatalysis research primarily focuses on hydrogen peroxide synthesis, neglecting process design and separation. Additionally, hydrogen peroxide’s thermodynamic instability in alkaline conditions and the existence of other ions make the separation difficult. This paper proposes a novel concept for the paired water electrolysis process that can be used to improve green hydrogen production economics through valuable chemical coproductions. Valorizing hydrogen peroxide to sodium percarbonate as the final product was chosen to address hydrogen peroxide separation challenges. An electrolyzer stack of 2 MW was chosen, incorporating a recirculating structure, and a boron-doped diamond anode to enhance the hydrogen peroxide production as the base case. According to the techno-economic analysis, for a 2 MW electrolyzer stack, capital expenditure was calculated as 64.5 M€, operational expenses as 21.6 M€, and revenue was calculated as 2.5 M€, resulting in a negative cash flow of −19.1 M€. Results revealed that the process can be profitable (breakeven point) at a capacity of approximately 308 electrolyzer stacks, which is 616 MW in capacity. A sensitivity analysis was conducted to determine how cost drivers including electricity price, anode price, Faradaic efficiency, price of the products and tax subsidy affect the breakeven point. A breakeven point of 60 electrolyzer stacks (120 MW) was found with a 100% increase in the sodium percarbonate sale price. In comparison, a theoretical 100% Faradaic efficiency in the anode material would result in a breakeven point of 38 electrolyzer stacks (76 MW). Even a more realistic 75% Faradaic efficiency leads to a breakeven plant size of 75 stacks (150 MW). Further, multiple two-parameter sensitivity analyses were conducted to assess the relations between Faradaic efficiency, sodium percarbonate sale price and anode material price. For instance, if sodium percarbonate price increases by 100% and Faradaic efficiency increases to 75%, the breakeven capacity drops down to 13 stacks (26 MW). Despite facing economic challenges for the proposed process design based on available technologies, the techno-economic analysis highlights key targets for future works. It also provides valuable insights into the economic feasibility of simultaneously producing hydrogen and sodium percarbonate through water electrolysis, indicating promising potential for the future.

## 1 Introduction

The issues of climate change related to greenhouse gas emissions have brought hydrogen (H_2_) sharply into focus as a critical solution for the energy transition. Additionally, H_2_ plays a significant role in the industry as a feedstock in various industrial processes, including ammonia, methanol, and steel production. Hydrogen is also a clean fuel that can be used for heating, thus enhancing its importance in fostering sustainability across a variety of sectors. Essentially, the significance of H_2_ and in particular, green hydrogen lies in its capability to assist in the transition toward cleaner energy systems, as well as revolutionize industrial processes, contributing to global energy security and environmental sustainability.^1−5^

The steam methane reforming process remains the predominant method of producing H_2_ worldwide (∼95%).^6^ In recent years, there has been a growing emphasis on producing green H_2_ through electrolysis using renewable energy sources, such as wind and solar power. Electrolysis technologies have gained considerable attention, particularly proton exchange membranes (PEM) and alkaline electrolysis, because of their potential to produce H_2_ without carbon emissions. However, the process economics are still not favorable, despite the promise of this approach. The price range of H_2_ obtained by water electrolysis (2.75–7.5 €/kg) is still not competitive with the price range of H_2_ obtained via steam methane reforming (0.5–1.5 €/kg).^7−11^

The relatively low market value of the coproduced oxygen (32 €/tonne)^9^ in water electrolysis is one of the economic disadvantages of the process. Recent studies have suggested that O_2_ production can be substituted for valuable chemical coproduction in water electrolysis to lower the H_2_ price. Studies have shown that anodic production of hydrogen peroxide (H_2_O_2_) is a viable coproduct of water electrolysis.^9,12−14^ Despite extensive literature coverage^15−19^ of cathodic H_2_O_2_ production via 2e^–^ oxygen reduction reaction (2e^–^ ORR), anodic H_2_O_2_ production through 2e^–^ water oxidation reaction (2e^–^ WOR) is less common due to the thermodynamic unfavorability of the pathway (due to the competing coproduction of O_2_). The 2e^–^ WOR technique is the focus of the current study since it represents a coveted electrochemical reaction pathway that can produce the valuable coproduct H_2_O_2_ while still producing green hydrogen, making it extremely desirable.^20,21^

Mavrikis et al. compiled a review of the proposed reaction mechanisms of 2e^–^ WOR, which are critical for the fabrication of electrocatalysts and the assembly of electrochemical reactors. Furthermore, different electrode materials and electrolytes were examined in their investigation.^20^ Recent developments in H_2_O_2_ production by water oxidation, including fundamentals, materials, strategies for increasing efficiency, etc. were reviewed by Xue et al. They discussed the challenges and future works for H_2_O_2_ production by water oxidation, including material stability, and industrial-scale analysis such as techno-economic evaluation.^22^ Anantharaj et al. identified the strategies employed in the design of catalysts for both 2e^–^ ORR and 2e^–^ WOR and proposed a few simple principles that have enabled the prediction of other prospective elements within the periodic table that can also form H_2_O_2_ selective catalysts.^23^ According to Perry et al., electrochemical H_2_O_2_ electrosynthesis technologies have the potential to challenge the conventional anthraquinone process to produce H_2_O_2_, but further progress is required before the electrochemical route will be able to compete. Based on examining the advances made in the fields of materials and reactor design, they concluded that further developments in materials are crucial for improving stability and production rates as well as reducing operating costs.^24^

Although novel 2e^–^ WOR electrocatalysts have shown significant promise at low electrical currents, their electrocatalytic capabilities decrease at larger current densities. Mavrikis et al. developed modulated boron-doped diamond (BDD) films which achieved an impressive 87% Faradaic efficiency (FE) and produced 76.4 μmol of H_2_O_2_/min/cm^2^ while maintaining stable electrochemical performance for 10 h at 200 mA/cm^2^. They also concluded that BDD is a viable candidate for the implementation of 2e^–^ WOR on a large scale.^25,26^ In another study, the electrochemical synthesis of H_2_O_2_ using BDD electrodes was reviewed by Espinoza-Montero et al. BDD electrodes were found to be promising materials for anodic H_2_O_2_ formation with their high FE and H_2_O_2_ production rate. However, the high cost of BDD electrodes may limit their application at an industrial scale.^27^ Using BDD as the anode, Pangotra et al. examined the effects of different parameters on the electrolyzer performance, including flow configuration, flow rate, and type of electrolyte. They achieved FE of up to 78% and a production rate of 79 μmol H_2_O_2_/min/cm^2^ as well as current densities of up to 700 mA/cm^2^ over a sustained period of 28 h.^28^

It has also been demonstrated that metal oxides and carbon-based electrodes can produce H_2_O_2_. Despite their impressive selectivity to H_2_O_2_, this study did not consider these materials because they have significantly low current density and short lifetimes, making their large-scale implementation questionable.^26,29,30^

H_2_O_2_ electrosynthesis relies heavily on an efficient product purification strategy, which has not yet been reported. Previous research has predominantly centered on the synthesis of H_2_O_2_, with no reported studies focusing on its separation. This omission is attributed to hydrogen peroxide’s thermodynamic instability when subjected to alkaline conditions.^31^ As a result, the separation process has been overlooked in favor of addressing challenges related to its synthesis, with even other techno-economic analyses treating the separation system as a black box.^9,32^ To the best of our knowledge and investigation, H_2_O_2_ separation from the electrolyte solution is extremely difficult. Qi et al. also proposed valorizing H_2_O_2_ into another valuable chemical due to the same separation problem.^31^

Since H_2_O_2_ and sodium carbonate (electrolyte) already exist in the process, as an alternative product, H_2_O_2_ can be valorized to produce sodium percarbonate (SPC). SPC (Na_2_CO_3_·1.5H_2_O_2_) is usually called solid H_2_O_2_ and is dissolved in water to produce H_2_O_2_. It has chemical properties similar to H_2_O_2_, is used as a bleaching agent in detergents, and is more efficient in several aspects. SPC can function over a wider pH range and offers a more economical alternative to H_2_O_2_ due to its solid form which makes it easier for transportation and storage.^33−36^ Furthermore, its market capacity in 2024 is estimated at 1.54 B€ and is projected to grow at 8.3% yearly.^37^

Although many studies have been conducted concerning the development of novel electrode materials, the evaluation of different types of electrolytes, the examination of reaction mechanisms, etc., the process design and techno-economic evaluation of large-scale industrial plants remain largely unexplored. This study presents a novel process design to produce H_2_O_2_ through water electrolysis and valorizing it into SPC as the final product, along with a detailed techno-economic analysis. Ultimately, a sensitivity analysis was conducted to assess how different parameters affect the economics of the process, including product prices, electrode prices, and FE.

## 2 Process Design

### 2.1 Coproduction of H_2_O_2_ and H_2_ in the Electrolyzer

**Figure 1:**
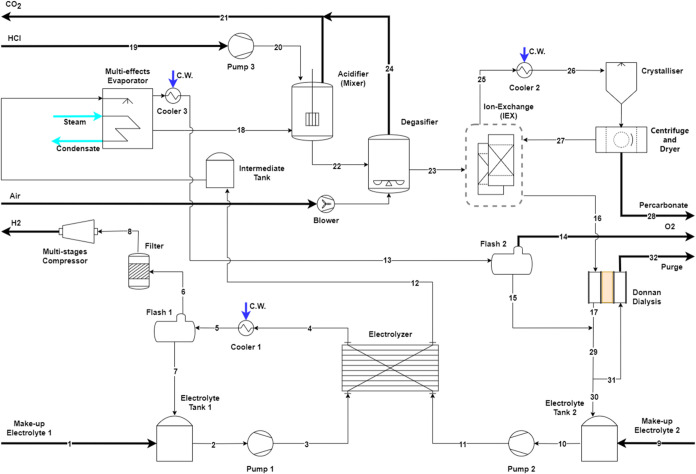
Process flow diagram of the proposed system. The catholyte recycles after a simple gas separation whereas the anolyte goes through a more complex chemical separation to extract SPC before recycling.

Figure 1 shows the proposed process design based on the electrochemical water oxidation reaction to H_2_O_2_ (2e^–^ WOR). The first part of the process involves the production of H_2_O_2_ through the electrolysis of water. Sodium carbonate (Na_2_CO_3_) is used as the electrolyte salt as it has been shown to improve FE toward the anodic H_2_O_2_ product.^38^ Sodium silicate is also added (in minor quantities) to improve the stability of the formed H_2_O_2_ in the process. Additionally, sodium hydroxide (NaOH) is periodically added to the electrolyzer to maintain the pH environment to promote H_2_O_2_ formation. The electrolyzer is maintained at ambient temperature with the help of cooling water. When a potential difference is applied across the cell, H_2_ and hydroxide ions are formed at the cathode via the water reduction reaction. The hydroxide ions migrate toward the anode and are oxidized to produce H_2_O_2_ and O_2_ at the anode. In addition to the significant amount of O_2_ that is produced directly at the anode, there is also considerable O_2_ produced through the decomposition of H_2_O_2_ at the anode surface and in the bulk electrolyte. The reactions are presented in Table 1.

**Table 1:** Electrochemical Reactions on the Anode and Cathode Sides

**Anode Reactions**		
1	2OH^–^ → H_2_O_2_ + 2e^–^	*E*_0_ = 1.78 V vs RHE
2	H_2_O_2_ + 2OH^–^ → O_2_ + 2H_2_O + 2e^–^	*E*_0_ = 0.69 V vs RHE
3	4OH^–^ → O_2_ + 2H_2_O + 4e^–^	*E*_0_ = 1.23 V vs RHE
**Cathode Reaction**		
1	2H_2_O + 2e^–^ → H_2_ + 2OH^–^	*E*_0_ = 0.00 V vs RHE

### 2.2 Electrolyzer Structure

Two types of flow structures were considered for the electrolyzer configuration: a single-pass flow system and a recirculating flow system. Single-pass flow systems continuously pass fresh electrolyte solution through the electrolyzer. At the electrolyzer outlet, the electrolyte is separated from products and recycled. Alternatively, the recirculating flow involves first recirculating the electrolyte solution within an electrolyzer until a desired concentration of H_2_O_2_ is reached. Following this, the electrolyte and products are separated. The discontinuous nature of the recirculating electrolyzer and the continuous nature of the separation can be managed through an intermediate holding tank.

The production rate is assumed to be constant for both structures since the electrolyzer capacity is fixed at 2 MW. However, the concentration of H_2_O_2_ achieved at the electrolyzer outlet is significantly lower in a single-pass structure than for the recirculating flow.^26,28^ This lower concentration will lead to a very difficult downstream separation. Thus, a recirculating structure was chosen despite the need for an extra tank. Following electrolysis, the anode and cathode outlets need to undergo a separation process to obtain products with the required specifications as well as recover the electrolyte.

### 2.3 H_2_ Separation

The cathode side separation is relatively easy as H_2_ is in the gas phase and the electrolyte solution is in the liquid phase. The cathode outlet is cooled and flashed to recover the H_2_, and the electrolyte is recirculated (Stream 4). The H_2_ is then compressed in a three-stage compressor to a final pressure of 50 bar and stored (Stream 8).

### 2.4 Challenges in H_2_O_2_ Separation

On the anode side, however, H_2_O_2_ and the electrolyte solution are in the liquid phase, which poses a difficulty in the separation. H_2_O_2_ is commercially separated by vacuum distillation in the anthraquinone process. However, in our case, the distillation of the electrolyte solution and H_2_O_2_ will result in water as the top product and the electrolyte salts and H_2_O_2_ as the bottom products. Attempting to separate small amounts of H_2_O_2_ from the electrolyte salts yields a product with very low purity. Even if it is assumed that the electrolyte salts can be perfectly removed from the solution, resulting in a pure water and H_2_O_2_ mixture before distillation, the energy required to concentrate the solution to the typical 30 wt % H_2_O_2_ is 109 kWh/kg product (based on Aspen Plus simulation). This significant energy demand, driven by the low concentrations in the electrolyzer outlet, is more than six times the aggregate energy consumption of the conventional anthraquinone process, which requires only 17.6 kWh/kg product to produce H_2_O_2_.^28^ To overcome these challenges, we are forced to consider other valorization methods. One such possible product is SPC made by complexing H_2_O_2_ with Na_2_CO_3_. In our process, the outlet stream of the electrolyzer consists of H_2_O_2_, Na_2_CO_3_, and water making SPC a feasible valorized product.

### 2.5 Design Basis and Assumptions

To begin designing the process, several assumptions were made, which are listed below:

1. All the design calculations are based on a 2 MW electrolyzer stack. This capacity is considered based on commercial electrolyzer stacks for producing H_2_.^39^
2. BDD was selected as the anode material because of its high FE and overall stability as mentioned above in this paper.
3. Based on the literature, all the calculations in this paper are based on a cell potential of 5 V, current density of 0.1 A/cm^2^, outlet H_2_O_2_ concentration of 6 × 10^–5^ kmol/kg and the FE toward H_2_O_2_ is 40%.^28,40^ The combination of the cell potential and current density is so chosen based on highest production of H_2_O_2_ at steady state.
4. The FE of the cathode toward H_2_ was assumed to be 100%.^41,42^
5. A well-controlled process with a fixed current density is assumed throughout the study, ensuring consistency in the electrolysis rate across various operational conditions.
6. The effect of sodium silicate was neglected in the process, and it was assumed to behave like sodium carbonate due to its similar chemical structure.
7. Considering the small quantity of NaOH added required to maintain anolyte pH, we do not include the small makeup stream in the design. Additionally, since the function of NaOH is pH maintenance, the OH^–^ ions are reacted away at the anode thus not affecting the process.
8. Sweden was selected as the process plant location because of cheap electricity prices as well as proximity to ports.^43^
9. The scope of the design is limited to the production of SPC and H_2_. The transport, waste management and auxiliary units are out of scope.

### 2.6 Valorization of Hydrogen Peroxide to Sodium Percarbonate

The anodic outlet stream of the electrolyzer (Stream 12) consisting of Na_2_CO_3_ electrolyte and H_2_O_2_ must be valorized to SPC. In the commercial production of SPC, H_2_O_2_ is mixed with sodium carbonate in a 1:1.0–1.5 molar ratio and crystallized.^44^ The reaction is as follows
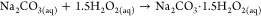
1

However, H_2_O_2_ and Na_2_CO_3_ are present in a molar ratio of approximately 1:17 in the electrolyzer’s outlet. Therefore, it is necessary to reduce the carbonate salt content. With the correct ratio, the commercial process involves the addition of sodium chloride (NaCl) to produce a salting-out effect for crystallization.

### 2.7 Excess Water Removal

Since the electrolyzer outlet is mostly water (Stream 12), in the first step, it is necessary to separate as much water as possible after the electrolyzer to reduce the downstream equipment sizing as well as increase the concentration of the product in the stream. A multi-effect evaporator was used at this step to remove large quantities of water in an energy-efficient and cost-effective manner. The evaporated water containing the oxygen (Stream 13) is sent to a flash vessel where the oxygen is separated from the water. The water is recirculated in the system (Stream 15), whereas the oxygen is vented out (Stream 14). Even though a 2 MW stack produces 72 kg/h of oxygen, it was found to be not economical to compress and sell it, and thus a decision was made to vent it. The concentrated solution of water, Na_2_CO_3_ electrolyte and H_2_O_2_ (Stream 18) is then sent for further processing.

### 2.8 Adjustment of pH

The water removal step is then followed by carbonate removal. In order to carry out an ion-based separation technique, the pH of the solution must be lowered to a value where H_2_O_2_ is not in its deprotonated form and only the electrolyte salt is in its ionic form. Thus, the pH of the solution is lowered from 13 (Stream 18) to 10 (Stream 22). This pH value is lower than the p*K*_a_ of H_2_O_2_ which is 11. Hydrochloric acid (Stream 19) was chosen as the appropriate acid primarily because it is a strong acid and can act as an additional source of Cl^–^ ions. The formation of water, NaCl salt, and carbon dioxide occurs upon adding this acid to the solution. Therefore, it is necessary to add a degasifier on Stream 22 following the acid treatment and before the ion-exchange resin to remove carbon dioxide. Stream 23, now adjusted for pH and free of carbon dioxide is sent downstream.

### 2.9 Excess Na_2_CO_3_ Removal

Following acid treatment, carbonate ions should be removed to reach the ideal ratio. Ion exchange is a method to exchange unwanted ions in water with other nonobjectionable ionic substances. A strong base anion exchange resin in the chloride form can remove the carbonate ions by exchanging one carbonate ion for two chloride ions.^45^ This process is reversible, and the resin can be regenerated with brine. The reaction is as follows (Z represents the resin)
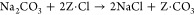
2

While other ion separation techniques such as electrodialysis and Donnan dialysis were considered, ion-exchange is most suitable here and was selected for the process design due to the following reasons: (1) It is easier in practice to implement compared to other considered technologies. (2) There is better control over how much carbonate is removed. (3) There is no additional NaCl salt required as it exchanges carbonate ions for chloride ions. (4) Pumping costs are the only energy requirement. (5) Resin can be regenerated to recover carbonate lost during operation.

Stream 23, containing water, Na_2_CO_3_ and H_2_O_2_ enters the ion-exchange unit and leaves carbonate free via Stream 28 for crystallization.

### 2.10 Sodium Percarbonate Formation and Crystallization

After reducing the carbonate content in water to achieve the desired molar ratio for the SPC reaction, the next step is to cool down the ion-exchange outlet stream and then crystallize the salt. Here, SPC is crystallized, separated, and dried. This step was considered similar to commercial SPC production.^44^ It was assumed that all the produced H_2_O_2_ is converted into SPC and all the produced SPC is recovered through crystallization (Stream 28). The leftover brine solution after the crystallization step (Stream 27) is used to regenerate the ion-exchange column, exchanging chloride for carbonate ions, to be recycled into the electrolyte tank (Stream 16). However, it is necessary to fully remove chloride ions from Stream 16 before recycling it to the electrolyzer. The excess chloride ions can be traced back to the addition of hydrochloric acid in the previous steps. To avoid chlorine gas formation and to recover carbonate from the purge stream, it was decided to employ Donnan dialysis. The draw solution was considered as the cleaned purge stream with water and Na_2_CO_3_ (Stream 31). There is an exchange of the carbonate and chlorine ions between Streams 16 and 31 within the apparatus. Thus, one of the outlets of the apparatus only consists of water and Na_2_CO_3_ (Stream 17) which is then recycled to the electrolyzer. The second outlet is a waste stream consisting of water and NaCl (Stream 32). However, the waste stream generated can be effectively managed by discharging it to a local water treatment facility via the municipal sewer system. This method offers a cost-effective solution, provided that the effluent composition complies with local regulations.^46^ Additional water and Na_2_CO_3_ need to be continuously provided to the process to make up for the water consumed in the electrolyzer, water lost in the purge stream, and Na_2_CO_3_ lost through the SPC product indicated via Steams 1 and 9.

It should be noted that flash drums, pumps, coolers, and the water evaporator were simulated using Aspen Plus software. All the other equipment calculations were done by hand. The separations in the degasifier, ion-exchange column, crystallizer, and Donnan dialysis were assumed perfect for ease of calculation. A detailed stream summary is presented in Table S1.

## 3 Techno-Economics

This section presents the detailed techno-economic calculation of the proposed process. First, a comprehensive list of equipment was compiled and approximately designed based on the process flow diagram. Capital expenditures (CAPEX) estimation was undertaken using factorial methods with an accuracy of ±35%.^47^ This method proves to be valuable at this project stage, where detailed engineering data is inaccessible, and approximating certain factors provides a rough estimate of the anticipated investment.

The approach employed was the Lang method.^48^ This method utilizes a Lang factor, which is an estimated ratio of the overall installation cost of the plant and equipment to the delivered equipment cost. The higher the Lang factor, the lower the impact of the equipment cost on the total installed cost and vice versa. The factor depends on the type of plant, equipment, and construction material.

Since the proposed process incorporates specialized equipment such as the electrolyzer, expensive anode material, and common materials like stainless steel, a decision was made to differentiate between various equipment categories with a Lang factor varying between 2 and 4. The first category is regular equipment, like pumps, storage tanks, the dryer, and the evaporator. The Lang factor considered for this category is 4, primarily due to the extra costs associated with the construction material. The second category, special equipment, includes the crystallizer, the ion-exchange unit, and the Donnan dialysis unit, with a Lang factor of 3. This factor accounts for both the expensive material and the auxiliary parts and equipment required for the unit. The third category pertains to electrical equipment, such as the electrolyzer and the cathode, with a Lang factor of 2. This lower factor is attributed to the installation’s emphasis on wiring rather than piping. An additional crucial consideration is the exceptionally expensive anode price, constituting nearly 75% of the total bare equipment cost. Due to the anode costs being related solely to the material price, the Lang factor for the anode is set to 1.

The bare equipment cost for the electrolyzer was calculated based on the area of the anode, cathode, membrane, and housing.^9^ The remaining equipment costs were calculated using the Matche cost estimator.^49^ The bare equipment costs are presented in Table S5. The complete breakdown of capital investment is shown in Table 2 where the total capital investment was calculated to be 64.52 M€.

**Table 2:** Total Capital Investment Calculation and Breakdown

	regular equipment	special equipment	electrolyzer	cathode	anode	total
equipment cost (M€)	4.089	1.168	0.054	0.003	14.579	19.893
lang factor	4	3	2	2	1	
installed equipment cost (M€)	16.354	3.505	0.108	0.006	14.582	34.555
OSBL cost (M€)	8.177	1.680	0.054	0.003	0.003	9.989
contingency (M€)	6.133	1.314	0.040	0.002	0.002	7.492
fixed capital investment (M€)	30.664	6.572	0.202	0.010	14.587	52.036
working capital (M€)	6.133	1.314	0.040	0.002	0.002	7.492
start-up cost (M€)	4.089	0.876	0.027	0.001	0.001	4.995
total capital investment (M€)	40.886	8.762	0.27	0.014	14.59	64.522

In general, operational expenditures (OPEX) refer to the day-to-day costs an organization incurs to maintain its regular operations, and some of the OPEX costs are dependent on the CAPEX costs. OPEX costs include direct production costs, capital expenditures, plant overhead and general expenses. Direct production costs include raw materials, utilities, equipment maintenance and repair, labor required for daily operations as well as patent costs. Capital charges were calculated for a 10-year lifetime of the project and at a 12% interest rate on the investment amount. Capital charge is the cost to the business for borrowing the required capital or in other words, it is the necessary return on the made investment. The 10-year lifetime is considered because the BDD anode is very expensive and can potentially last up to 10 years without needing replacement.^9^ The 12% interest rate was decided based on investment risks and inflation. Plant overhead was defined as the total cost involved in operations which accounts for operational efficiency and the well-being of the facility (safety, recreation, and laboratories). This cannot be traced directly to the product. Additionally, other fixed and variable general expenses were considered. Fixed expenses include sales and marketing, engineering services and R&D. Variable costs include distribution, training, and management. Operational expenditures amounted to 21.59 M€/yr. Table 3 shows the cost breakdown of OPEX. A detailed breakdown is presented in Tables S2 to S4.

**Table 3:** Complete Operating Costs Calculation and Breakdown

category	M€/yr
**direct production costs**	**6.10**
fixed costs	0.05
variable costs	6.05
**capital charges**	**11.42**
**plant overhead**	**0.68**
**general expenses**	**3.39**
fixed (sales & marketing, engineering, R&D)	1.36
variable (distribution, training, management)	2.03
**total manufacturing costs (TMC)**	**21.59**

The annual revenue generated by selling hydrogen and SPC produced by a 2 MW stack with prices of 5000 €/tonne and 780 €/tonne, respectively, was found to be 2.54 M€. Based on the annual revenue and the calculated OPEX, the annual cash flow was estimated. A negative annual cash flow was calculated for the designed process at −19.05 M€/yr. The main cost driver of OPEX is the cost of capital (53% of OPEX) which is majorly driven by the expensive anode material. Therefore, with current equipment efficiencies and prices for raw materials, utilities and final products, a one-stack electrolyzer project is considered unprofitable. However, it is considered that for larger capacity projects, operating costs will not increase linearly, hence a breakeven analysis was conducted.

A breakeven analysis was performed on the project’s production capacity, according to the six-tenths heuristic. According to this heuristic, if the cost of a given unit at one capacity is known, the cost of a similar unit at X times the capacity of the first is calculated by the following formula
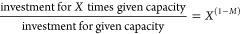
3

**Figure 2:**
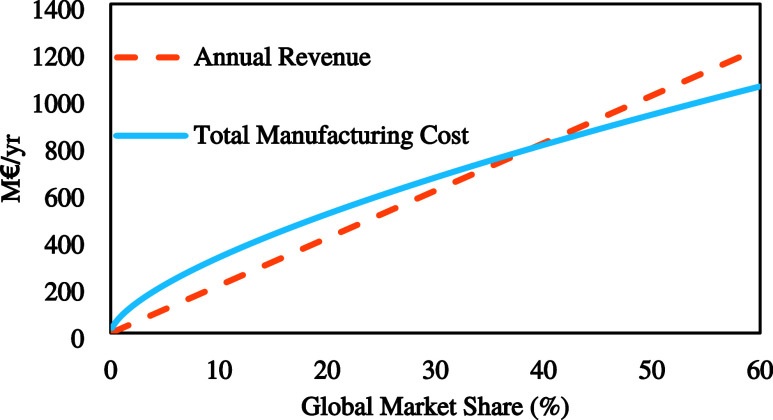
Breakeven point analysis based on a six-tenths heuristic for scaling the plant capacity.

For all equipment except the electrolyzer, it is considered that the scaling-up costs are minimal, therefore factor *M* was given the value of 0.4. However, assuming that the electrolyzer is a modular piece of equipment, *M* was considered 0.2 for the electrolyzer, the anode, and the cathode. For capacities of up to 60% of the global SPC market (considering a 1.54 B€ market capacity), the breakeven analysis is shown in Figure 2. According to the figure, the breakeven point at which the project is considered profitable was found at a capacity equivalent to 39% of the global market. This market share translates to a process operating 308 electrolyzer stacks (2 MW each). It is important to note that the capacity serves only as an indicator and not a possible scenario, since the global market distribution can be a limiting factor on it.

Further economic analysis was conducted at a capacity of 339 stacks (10% higher than the breakeven point) including Net Present Value (NPV) and Internal Rate of Return (IRR). IRR represents the discount rate at which the NPV of an investment becomes zero. A higher IRR typically indicates a more attractive investment opportunity. The NPV for a lifetime of 10 years was calculated based on annual operating expenses, expected depreciation, and taxes. The NPV at the end of 10 years was negative, indicating that this process as a business proposition is not profitable in its current form. Based on cash flows for 10 years since the commencement of the plant, the IRR was 7.92%. Even though at this scale the IRR is positive, it is not large enough to yield a positive NPV, indicating an economically unfavorable investment.

**Figure 3:**
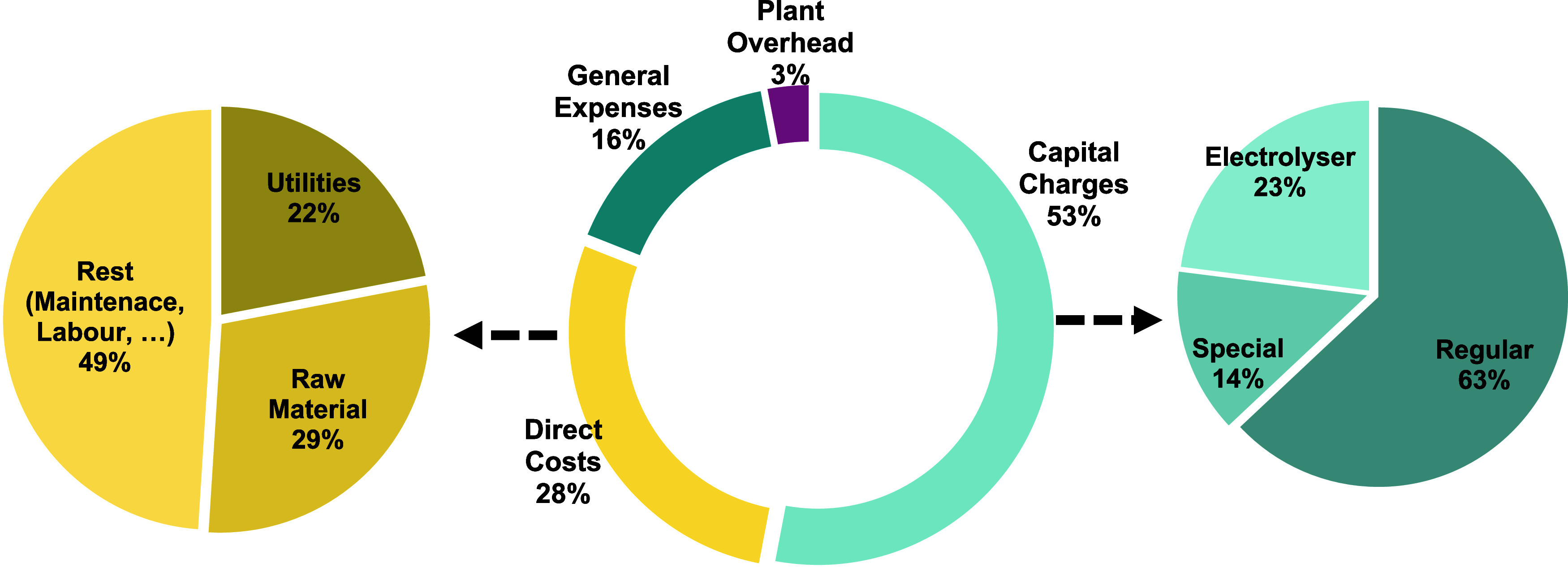
Cost review of the TMC.

Thus, it was deemed critical to analyze the factors that affect these costs the most and understand if and how this design can be made into an economically feasible project. In Figure 3, the cost review is depicted, leading to the need for a sensitivity analysis of the most impactful parameters.

## 4 Sensitivity Analysis

Sensitivity analysis evaluates the effect of changes in input parameters on a model’s or system’s output. This analysis allows one to identify the pivotal factors of a system or process. This can assist future studies by focusing on the most critical factors. This study focused on the cost drivers’ impacts on the process economics. In our case, the main cost drivers investigated are electricity price, anode price, FE, product prices and tax subsidies. To gain a comprehensive understanding of the impact of cost drivers, two different sensitivity analyses were conducted: (1) Breakeven Point Analysis and (2) NPV and IRR Analyses.

### 4.1 Breakeven Point Analysis

Earlier in the techno-economics section, it was noted that the breakeven point for the process is 308 stacks, which represents 616 MW of plant capacity and 39% of the worldwide market for SPC, which is considered an unreasonable scale. In this section, we investigate the influence of cost drivers on the breakeven point to identify targets that can make this process more economically feasible.

#### 4.1.1 Electricity Price

**Figure 4:**
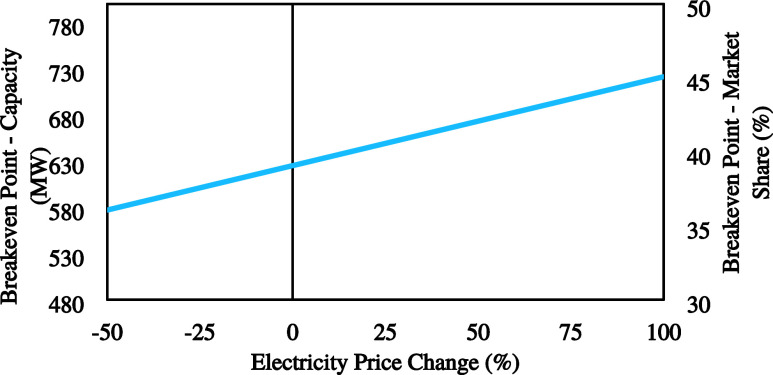
Effect of the electricity price on the breakeven point.

The electricity price is one of the key parameters that can change throughout the year and have a significant impact on process economics. Electricity prices historically have shown great volatility which could result in high uncertainties for the operating expenses, and therefore, for the overall economics of the process. In the past five years, electricity prices have spiked up to approximately 10 times higher than the average, due to extreme circumstances, and on average they fluctuate between −50% and +100% from today’s prices (30–120 €/MWh).^43^ As a result, a sensitivity analysis was conducted using this specific range of values. In Figure 4, the breakeven point changes are depicted in relation to variations in the electricity price. In the case of a 50% reduction in the electricity price, the breakeven point for the global market share of SPC decreases approximately by 5% from around 40% to about 35%. While electricity prices have an enormous impact on a standard alkaline water electrolysis plant’s economic viability, they affect this process very little.

#### 4.1.2 Anode Price

**Figure 5:**
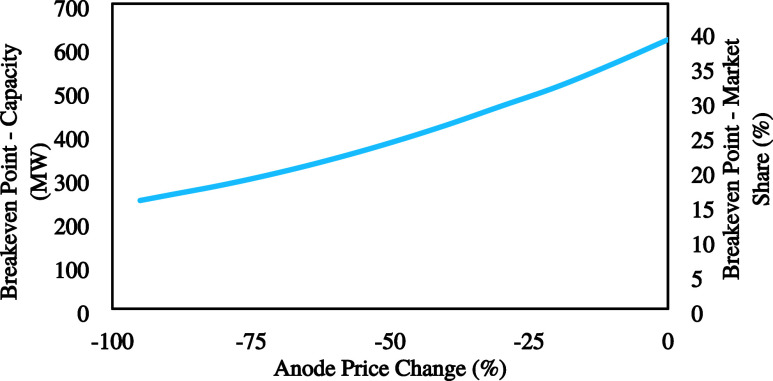
Effect of the anode price on the breakeven point.

One of the most important factors affecting CAPEX is the anode cost. This high cost is attributed to the current manufacturing technology. Advancements in material science and manufacturing technology could lead to the development of alternative materials (not necessarily BDD) with comparable or higher FE at significantly reduced costs. Thus, by reducing the anode cost from 0 to 95%, its effect on the breakeven point was investigated. The breakeven point changes with the decreasing anode price shown in Figure 5.

Results show a significant change in plant capacity and breakeven point by changing the anode cost. A 95% reduction in the anode price leads to a breakeven point of around 15% of the global SPC market share which is a reasonable capacity. However, it could be challenging to develop a novel, stable and cheap anode for this process in the coming years.

#### 4.1.3 Faradaic Efficiency

Faradaic efficiency in electrolysis is of paramount importance as it directly influences the efficacy of electrochemical processes crucial for sustainable energy technologies. It measures the extent to which the desired electrochemical reactions occur, ensuring that energy is efficiently converted without wasteful side reactions. A high FE signifies an optimized electrolysis process, minimizing energy losses and maximizing the yield of the intended products.

This section aims to identify the effect of FE on the economic breakeven points. A sensitivity analysis was done on the FE range of 40% to an ideal case of 100%.

There are some assumptions for this analysis below:

- Although the percarbonate production increases when the efficiency is increased, the total amount of product is significantly lower than the electrolyte flow rate, resulting in the same equipment size (constant CAPEX).
- In terms of OPEX, the only difference is related to the sodium carbonate consumed in the production of SPC.

**Figure 6:**
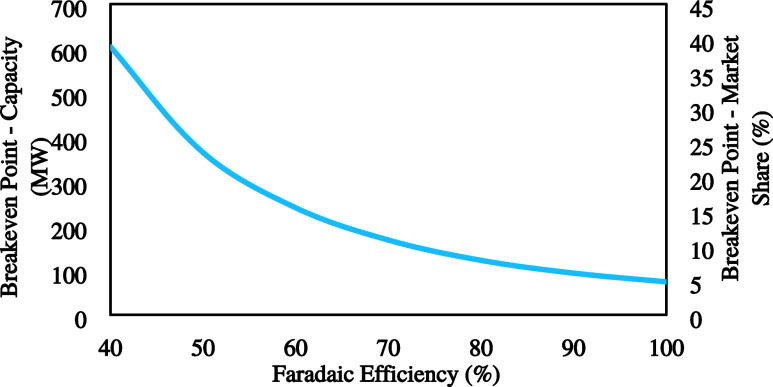
Effect of the Faradaic efficiency on the breakeven point.

Figure 6 presents the market share and the capacity that should be targeted to make the process economically feasible as a function of FE. Based on the current technology level (FE of around 40%), to reach a breakeven point, a plant size of 616 MW (39% of the market) should be targeted. However, in the ideal case (FE of 100%), market share and plant size targets can be significantly lowered to around 5% and 76 MW, respectively, to reach the breakeven point.

As previously indicated, due to the low concentration of product at the electrolyzer outlet, the separation stays the same by increasing the FE (same CAPEX). The increase in FE, however, directly increases revenue (production rate), while only OPEX changes. Therefore, FE was found to be the most important factor in this techno-economic analysis. Furthermore, anodes with high FE of ∼80% have already been demonstrated at lab scales, indicating that this scenario is not highly unlikely to take place.^25,26^

#### 4.1.4 Product Prices

Hydrogen and SPC prices may change due to market fluctuations. Thus, two scenarios were considered for performing the analysis:

- Scenario 1: Hydrogen price remains constant (5000 €/tonne) while SPC price changes from −50 to +100% (390 to 1560 €/tonne)
- Scenario 2: SPC price remains constant (780 €/tonne) while hydrogen price changes from −50 to +100% (2500 to 10,000 €/tonne)

**Figure 7:**
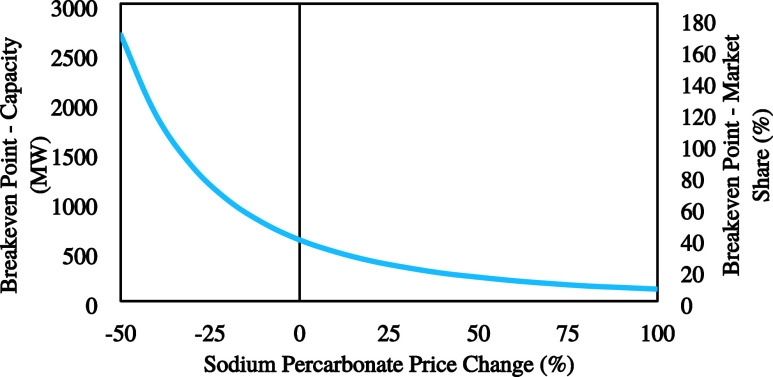
Effect of the SPC price on the breakeven point.

**Figure 8:**
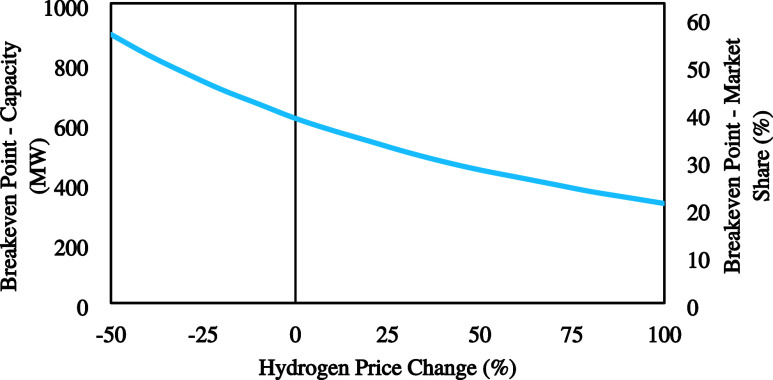
Effect of the hydrogen price on the breakeven point.

Figures 7 and 8 represent the breakeven point changes for scenario 1 and scenario 2, respectively.

It was calculated that if the SPC price is doubled, the breakeven point drops to almost 7.5% of the global SPC market share, whereas if the hydrogen price is doubled, the breakeven point is almost 20%. Due to the larger mass of SPC produced compared to the mass of H_2_ produced, the impact of changing percarbonate sale price is greater. However, it should be noted that neither price will remain constant, and both prices will fluctuate in the long run. An increase in the price of both products will benefit the current design.

### 4.2 NPV and IRR Analyses

To determine the effect of cost drivers on NPV and IRR, a sensitivity analysis was performed. This analysis was conducted based on a realistic plant capacity of 200 MW (100 stacks) to ensure a fair comparison with the previous chapters’ base analysis. For the chosen capacity, NPV and IRR were calculated as −806 M€ and −5.84%, respectively.

#### 4.2.1 Electricity Price

**Figure 9:**
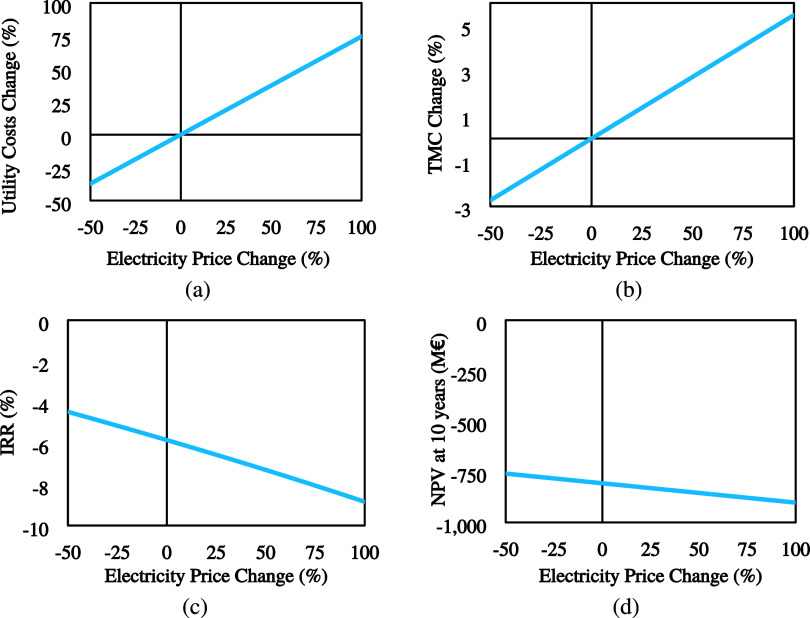
Effect of the electricity price on (a) Utility Costs, (b) TMC, (c) IRR, and (d) NPV.

In this section, several parameters were investigated, including utility costs, manufacturing costs, and overall process economics (NPV and IRR). Figure 9(a) shows the impact on utility costs. In extreme conditions, utility costs may vary between −37 and +75%, indicating a relatively high impact of the electricity price on utility costs. Figure 9(b) shows the impact on total manufacturing costs (TMC) is much lower than utility costs. For a −50 to +100% change in the electricity price, the change in TMC varies from −3 to +5%. Finally, the impact on the overall economics of the process is depicted in Figure 9(c),9(d). NPV and IRR increase steadily as the electricity price decreases, reaching −758 M€ and −4.46%, respectively. On the other hand, for a 100% increase in the electricity price, the NPV decreases to −902 M€ and IRR to −8.84%.

#### 4.2.2 Anode Price

**Figure 10:**
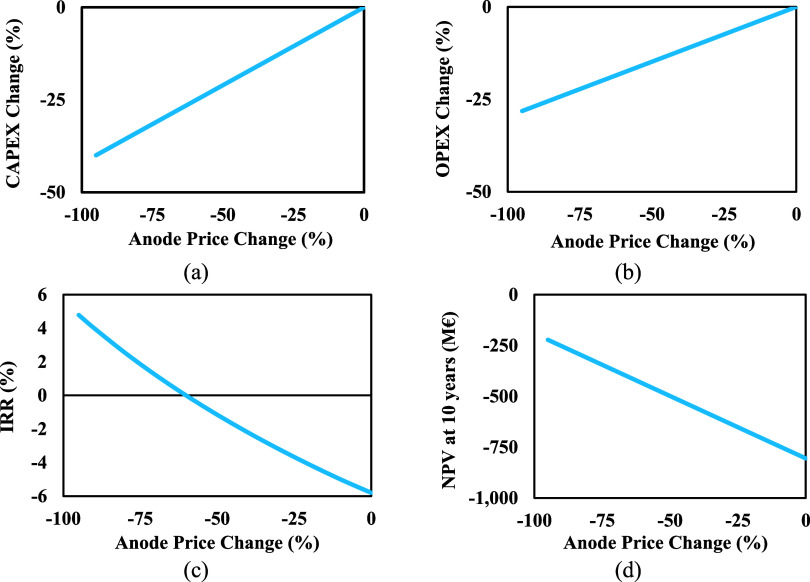
Effect of the anode price on (a) CAPEX, (b) OPEX, (c) IRR, and (d) NPV.

In a similar manner to how reducing the anode price affects the breakeven point, an analysis was conducted to evaluate the impact of reducing anode costs on CAPEX, OPEX, NPV, and IRR. During the reduction of anode cost from 0% to 95%, CAPEX can decline up to around 40% as seen in Figure 10(a). There is a linear relationship between CAPEX changes and reductions in anode costs. It can be seen in Figure 10(b) how changes in CAPEX result in changes in capital charge, which in turn leads to changes in OPEX. As shown in Figure 10(c),10(d), the changing CAPEX and OPEX have a significant impact on IRR and NPV. It can be seen that even with a 95% reduction in the anode cost, the IRR is 4.79% and the NPV is negative at −222 M€. This indicates that along with anode cost reduction, other parameters must change favorably to consider this project an acceptable investment.

#### 4.2.3 Faradaic Efficiency

In this section, the impact of FE as a key cost driver for the process on the process economy (NPV and IRR) was evaluated through sensitivity analysis. We conducted sensitivity studies in the range of 40% to the ideal case (efficiency equal to 100%) based on the FE of the current electrolyzers.

**Figure 11:**
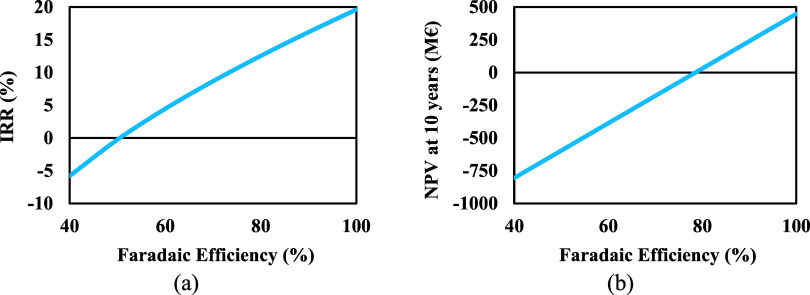
Effect of the Faradaic efficiency on (a) IRR, and (b) NPV.

Accordingly, assuming an ideal scenario, where the FE is 100%, the IRR and NPV are 19.62% and 447 M€, respectively. Even if a more feasible efficiency of 75% is considered as a target for the future, it is also shown that IRR and NPV can be significantly increased to approximately 10.60% and −75 M€ compared to around only −5.84% and −806 M€ currently at 40% FE (Figure 11).

#### 4.2.4 Product Prices

In this section, a sensitivity analysis was conducted on IRR and NPV for the same scenarios discussed in Section 4.1.4.

**Figure 12:**
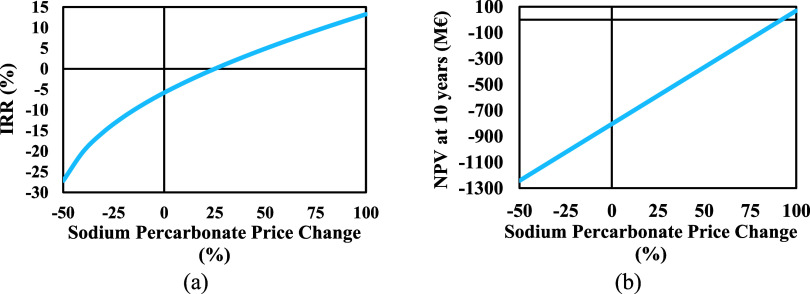
Effect of the sodium percarbonate price on (a) IRR, and (b) NPV.

For scenario 1, the IRR and the NPV can be seen in Figure 12(a),12(b). It can be seen that if SPC is sold at a 100% higher price, the IRR is 13.24% with a positive NPV of 69.44 M€ after 10 years. This is a plausible scenario considering this compound finds growing applications in cleaning powder and laundry detergents as an eco-friendly alternative to chlorine and can be marketed as a green product.^50^

**Figure 13:**
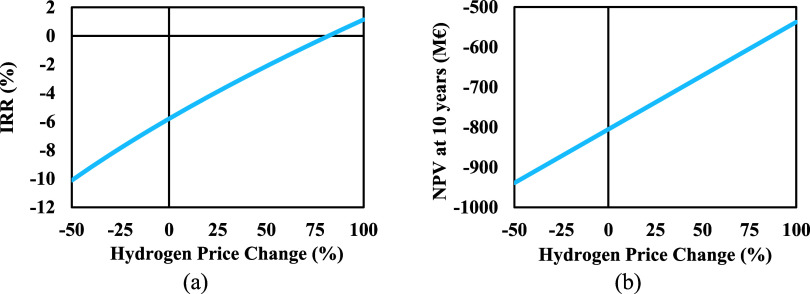
Effect of the hydrogen price on (a) IRR, and (b) NPV.

Figure 13(a),13(b) show the IRR and the NPV for scenario 2. Considering the increased demand for hydrogen, it will be possible if the hydrogen can be sold at a 100% higher price, however, the resulting IRR is approximately 2% with a negative NPV after 10 years.

There is a greater impact on the IRR and NPV when it comes to SPC price changes since the production rate by mass is far higher than hydrogen. However, the best-case scenario would be if both products were sold at a higher price.

#### 4.2.5 Tax Subsidy

To promote green hydrogen production, the Clean Hydrogen Production Tax Credit is one of the most important policies. This tax credit is intended to stimulate the domestic clean hydrogen industry and reduce the cost gap between green and fossil-based hydrogen. Assuming the project could be part of a green transition policy, the tax rate could drop from 20.6% to zero. A sensitivity analysis was conducted to examine how different tax rates affect the project economy. It is evident that earnings after tax, and consequently the IRR and NPV, will vary depending on tax rates.

**Figure 14:**
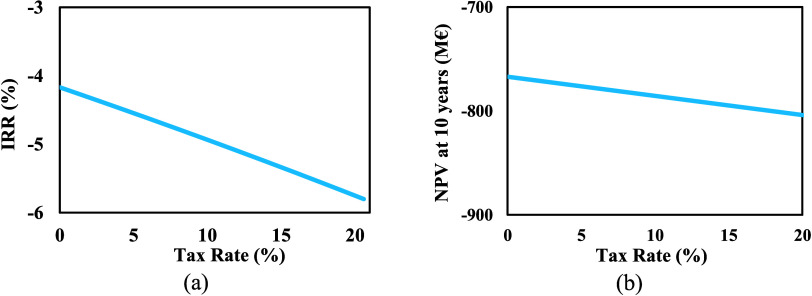
Effect of tax subsidy on (a) IRR, and (b) NPV.

Figure 14(a),14(b) illustrate that NPV and IRR increase steadily as the tax rate decreases, reaching −767 M€ and −4.17%, respectively. This indicates that tax subsidies do not have a significant influence on the project’s economic viability.

### 4.3 Overview of the Sensitivity Analysis

The sensitivity analysis demonstrated how the project’s economic performance varies with changing cost drivers. Some of these cost drivers have more impact than others. Table 4 illustrates how IRR and NPV are affected by changing cost drivers to their extreme values. The extreme values are ordered based on their positive effect on the economics, meaning that the most favorable scenarios were compared with each other.

**Table 4:** Comparison of the most favorable scenarios

scenarios	IRR (%)	NPV (M€)
base case	–5.8	–805
100% Faradaic efficiency	19.62	447.39
100% percarbonate price increase	13.24	69.43
95% anode price reduction	4.79	–222.01
100% hydrogen price increase	1.14	–537.81
100% tax subsidies	–4.21	–768.66
50% electricity price reduction	–4.46	–758.45

Based on Table 4, the cost drivers are ranked in order of their effectiveness power as follows: FE, SPC price, anode price, hydrogen price, tax subsidies, and electricity price.

### 4.4 Multiparameter analysis

The above analysis describes well how the process economics are affected by changing a single parameter while keeping the others constant. However, it rarely so happens that only one parameter changes. Thus, a two-parameter sensitivity analysis was performed on key parameters identified in the above section. The results are described in the following sections.

#### 4.4.1 Effect of Changing Faradaic Efficiency and SPC Price

**Figure 15:**
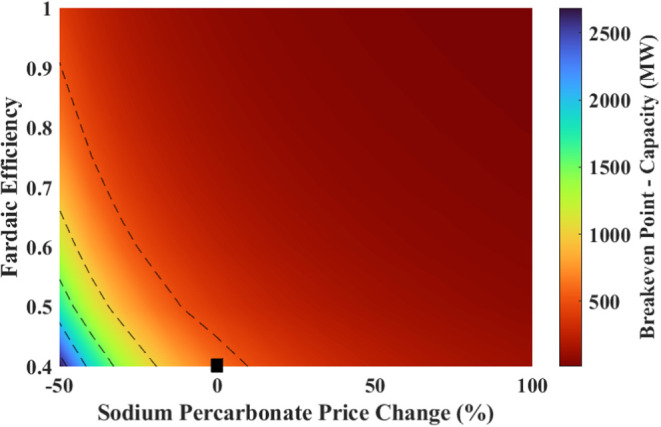
Effect of changes in the sodium percarbonate price and the Faradaic efficiency on the breakeven point (black marker shows the current situation).

**Figure 16:**
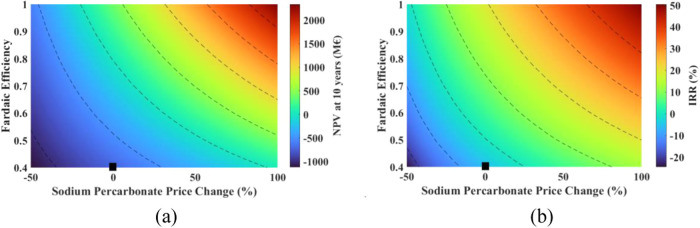
Effect of changes in the sodium percarbonate price and the Faradaic efficiency on (a) NPV, and (b) IRR (black marker shows the current situation).

Keeping in mind the most influential and likely parameters to change, a two-parameter sensitivity analysis was performed on the breakeven point, NPV and IRR by changing the FE and the SPC price. The results are shown in Figures 15 and 16.

The breakeven point improves drastically with a positive change in the FE and SPC price. With a 75% FE, the breakeven point ranges from 74 stacks (148 MW) to 13 stacks (26 MW) for a 0 to 100% increase in the price of SPC.

It can be seen that with the improvement in FE and an increase in SPC price, the process is economically more favorable. As seen in Figure 16, if FE can be improved to 75% and SPC can be sold at a price 0 to 100% more than the current cost, NPV ranges from −75 to 1383 M€ with IRR ranging from 10.6 to 35.8%.

#### 4.4.2 Effect of Changing Anode Price and SPC Price

**Figure 17:**
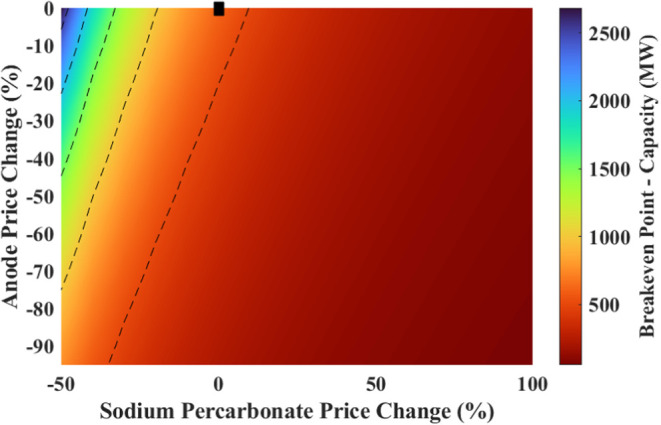
Effect of changes in the sodium percarbonate price and the anode price on the breakeven point (black marker shows the current situation).

**Figure 18:**
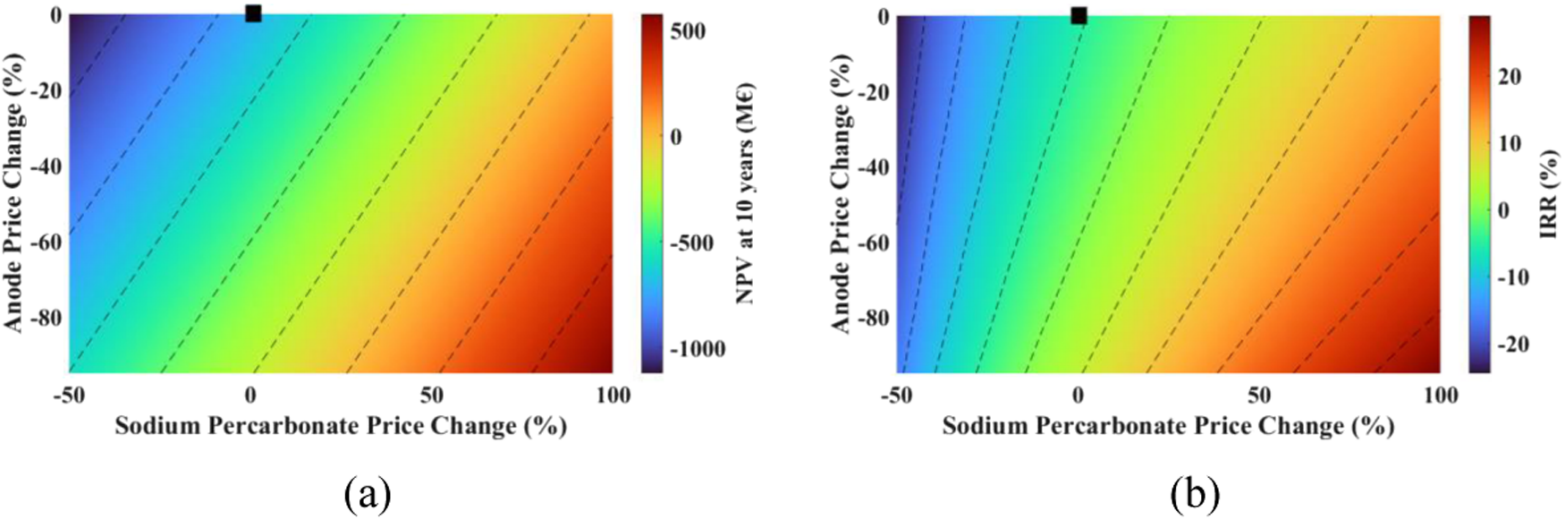
Effect of changes in the sodium percarbonate price and the anode price on (a) NPV, and (b) IRR (black marker shows the current situation).

Another two-way sensitivity analysis was performed on the breakeven point, NPV and IRR by changing the anode price and the SPC price. The results are shown in Figures 17 and 18.

The breakeven point improves with a positive change in the anode price and SPC price as well. With an 80% decrease in the anode price, the breakeven point ranges from 132 stacks (264 MW) to 31 stacks (62 MW) for a 0 to 100% increase in the price of SPC.

If the anode price decreases by 80% and SPC can be sold at a price 0 to 100% more than the current cost, NPV ranges from −290 M€ to 490 M€ with IRR ranging from 2.48 to 25.38%.

#### 4.4.3 Effect of Changing Anode Price and Faradaic Efficiency

**Figure 19:**
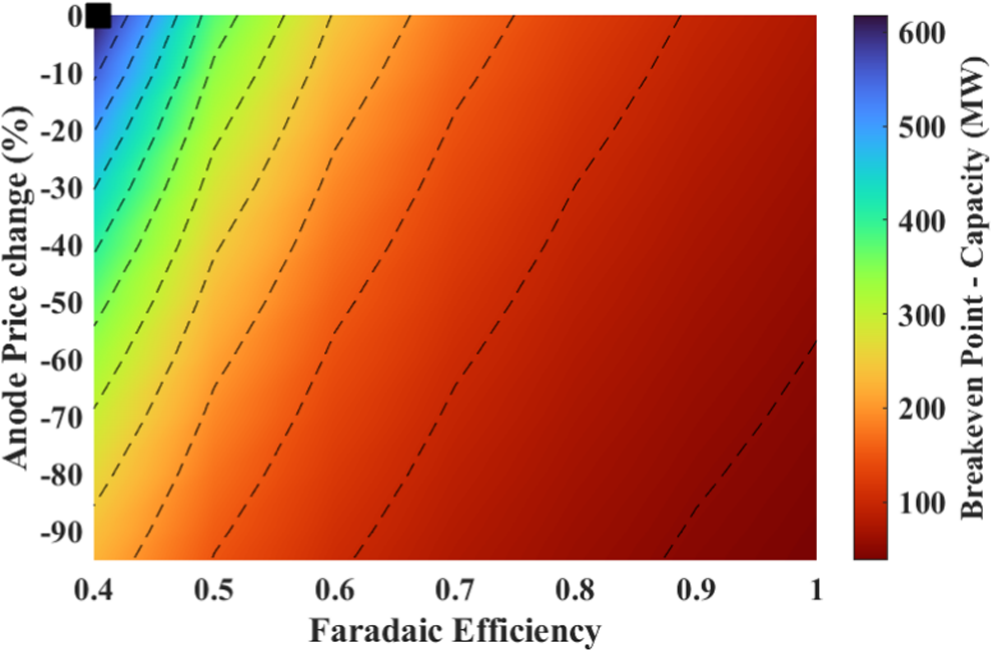
Effect of changes in the Faradaic efficiency and the anode price on the breakeven point (black marker shows the current situation).

**Figure 20:**
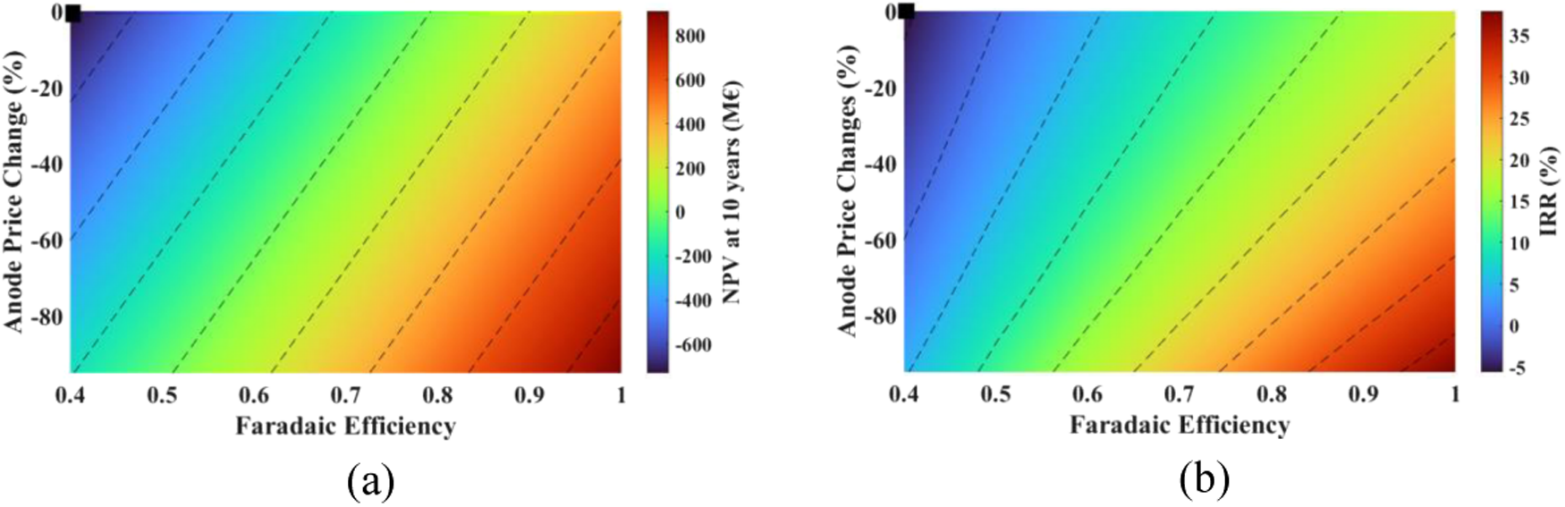
Effect of changes in Faradaic efficiency and the anode price on (a) NPV, and (b) IRR (black marker shows the current situation).

Multiparameters analysis was also performed on the breakeven point, NPV and IRR by changing the anode price and Faradaic efficiency. The results are shown in Figures 19 and 20.

The breakeven point benefits from a decrease in anode price and an increase in Faradaic efficiency. When the anode price drops by 80%, the breakeven point spans from 132 stacks (264 MW) to 21 stacks (42 MW), depending on Faradaic efficiency, which varies between 40 and 100%.

Considering that the anode price reduces by 80% and the Faradaic efficiency ranges between 0 and 100%, the NPV ranges from −290 to 827 M€, with an IRR ranging from 2.47 to 33.67% as shown in Figure 20.

#### 4.4.4 Overview of the Multiparameter Sensitivity Analysis

The multiparameter sensitivity analysis results highlight the positive impact on economics when two parameters change favorably. As illustrated by the figures provided, attaining a lower breakeven point is comparatively easier and more feasible. With a promising change in multiple variables, NPV tends toward the positive side after 10 years with an IRR value that is more attractive to investors.

### 4.5 Scenarios’ Likelihood Evaluation

It is considered that these scenarios would not occur with the same likelihood. According to the literature, it may be very likely that the FE will increase in the near future. Hydrogen and SPC prices may rise in the future due to high market demand, underestimation of the price in the current study, and the trend toward green technology. This scenario is very likely, as it reflects the current and projected situation of the hydrogen market. It also reflects the environmental and social factors that influence consumers’ preferences and choices.

On the other hand, the electricity price scenario is not very likely, as it faces many challenges and uncertainties, such as the reliability and availability of renewable energy, the cost and efficiency of energy storage, and the market and political forces that affect the energy sector. The anode price scenario is also not very likely soon, either, as it requires significant investment and research in material science and the overcoming of technical and operational difficulties.

Assessing the likelihood of tax subsidies is challenging, as they are highly dependent on the plant’s location, the prevailing policies, and the broader political landscape. In practice, tax rates may exceed the assumptions made in this study or fall within the considered range. However, their impact on the overall economics is relatively minor compared to other key parameters.

## 5 Conclusions

The purpose of this work was to bridge the gap between academic studies and the practical challenges regarding the coproduction of H_2_O_2_ via water electrolysis. The high cost of hydrogen production via water electrolysis led to some studies recommending the substitution of oxygen production for H_2_O_2_, which has a higher value. However, no studies explored the feasibility and costs of extracting H_2_O_2_ from the electrolyte. Hydrogen peroxide separation was found to be extremely difficult due to the presence of other ions that cannot be easily extracted. Thus, this study presented a novel conceptual process design as well as a techno-economic evaluation for the valorization of H_2_O_2_ into SPC. This decision was driven by the market feasibility of SPC and the availability of necessary raw materials in the electrolyzer outlet stream.

For the plant location Sweden was chosen, leveraging its low electricity costs, market accessibility, and port availability. The plant’s capacity was selected based on a modular 2 MW electrolyzer stack, employing a recirculating structure to achieve higher H_2_O_2_ concentrations. A boron-doped diamond anode was selected due to its better performance at high current densities and extended lifetime.

An economic analysis was conducted, and results revealed a projected CAPEX of 64.52 M€ and an annual OPEX of 21.59 M€ for a 2 MW electrolyzer stack (production rate of 2.5 ktonnes/yr of sodium percarbonate). With an anticipated annual revenue of 2.54 M€ per stack, a capacity of 308 stacks (616 MW) was required to breakeven with operating expenditure, representing a substantial portion of the global SPC market share (39%).

A more detailed economic evaluation of IRR and NPV was carried out considering a more realistic capacity of 100 stacks (200 MW) also revealed less favorable results. With a CAPEX of 3.2 B€ and OPEX of 870 M€, IRR was 7.92%, and NPV after 10 years was −509.25 M€.

While the proposed process design faces economic challenges using existing technologies, further exploration was carried out through sensitivity analysis to highlight the key targets for future advancements. It was seen that adjusting cost drivers including product prices, electricity price, anode price, and FE can lead to a significant reduction in the breakeven point as well as a considerable increase in IRR and NPV. FE followed by anode price were found to be the most crucial parameters. An ideal FE of 100% resulted in around 38% IRR and 3.8 B€ NPV. These numbers were calculated to be around 25% and 1 B€, respectively, for the 95% reduction in anode price. The electricity price, however, was found to be the least influential parameter, contrary to what is the case for alkaline water electrolysis. The multiparameter sensitivity analysis demonstrated that favorable change in multiple cost drivers has a significant impact on the economics. For a 0 to 100% increase in the price of SPC, with an 80% decrease in the anode price, the NPV ranges from −290 to 490 M€ with IRR ranging from 2.48 to 25.38%, whereas, with an increase from 40% to 75% FE, the NPV ranges from −75 to 1383 M€ with IRR ranging from 10.6% to 35.8%.

While sensitivity analysis and adjustments to individual and multiple parameters can be used to enhance the economic viability of the process, it is crucial to note that there is room for additional exploration and optimization. First, delving deeper into the analysis of critical parameters, particularly focusing on the influence of raw material prices and the utilization of chemical stabilizers. Additionally, since the economic analysis involves assumptions to reduce complexity, it is suggested to consider a broader spectrum of economic indicators related to the efficiency and sustainability of the process. Finally, it is recommended to investigate the current market dynamics to understand whether sodium percarbonate can also replace other products in the market and investigate whether there are new applications which can leverage the overall market capitalization.

## Supporting Information Available

Stream summary table; material and energy prices; operational expenses breakdown; operational expenses per unit; equipment list and cost (PDF)
