# Supplementary material for: Valorization of Hydrogen Peroxide for Sodium Percarbonate and Hydrogen Coproduction via Alkaline Water Electrolysis: Conceptual Process Design and Techno-Economic Evaluation
Source: Ind Eng Chem Res. 2025 Jan 24;64(5):2801–15. doi: 10.1021/acs.iecr.4c03408 (PMC11812344; doi:10.1021/acs.iecr.4c03408)
Supplement: Supplementary file 1 — ie4c03408_si_001.pdf [file ie4c03408_si_001.pdf]

Valorisation of Hydrogen Peroxide for Sodium Percarbonate and Hydrogen Co-production via Alkaline Water Electrolysis: Conceptual Process Design and Techno-Economic Evaluation

Mahdi Mohajeri [a,1], *Shachi Shanbhag* [a,1,*], *Eleftherios Trasias* [a,1], *Farzad Mousazadeh* [a], *Wiebren de Jong* [b], *Sohan A. Phadke* [b]

[a] Chemical Engineering Department, Delft University of Technology, Delft, 2629 HZ, The Netherlands

[b] Process and Energy Department, Delft University of Technology, Delft, 2628 CB, The Netherlands

[*] Corresponding author at: Chemical Engineering Department, Delft University of Technology, Delft, The Netherlands. Email: s.m.shanbhag@tudelft.nl

[1] These authors contributed equally to this work.

Table S1: Stream Summary Table

Stream no.	Water	Hydrogen	Hydrogen peroxide	Sodium Carbonate	Oxygen	HCL	CO2	NaCl	Sodium Percarbonate	Total
1	268.710	0.000	0.000	0.000	0.000	0.000	0.000	0.000	0.000	**268.710**
2	49638.986	0.000	0.000	0.000	0.000	0.000	0.000	0.000	0.000	**49638.986**
3	49638.986	0.000	0.000	`	0.000	0.000	0.000	0.000	0.000	**49638.986**
4	49370.276	14.916	0.000	0.000	0.000	0.000	0.000	0.000	0.000	**49385.192**
5	49370.276	14.916	0.000	0.000	0.000	0.000	0.000	0.000	0.000	**49385.192**
6	0.000	14.916	0.000	0.000	0.000	0.000	0.000	0.000	0.000	**14.916**
7	49370.276	0.000	0.000	0.000	0.000	0.000	0.000	0.000	0.000	**49370.276**
8	0.000	14.916	0.000	0.000	0.000	0.000	0.000	0.000	0.000	**14.916**
9	2454.388	0.000	0.000	512.420	0.000	0.000	0.000	0.000	0.000	**2966.807**
10	49638.986	0.000	0.000	5262.000	0.000	0.000	0.000	0.000	0.000	**54900.986**
11	49638.986	0.000	0.000	5262.000	0.000	0.000	0.000	0.000	0.000	**54900.986**
12	49719.599	0.000	101.473	5262.000	71.596	0.000	0.000	0.000	0.000	**55154.668**
13	35000.000	0.000	0.000	0.000	71.596	0.000	0.000	0.000	0.000	**35071.596**
14	0.000	0.000	0.000	0.000	71.596	0.000	0.000	0.000	0.000	**71.596**
15	35000.000	0.000	0.000	0.000	0.000	0.000	0.000	0.000	0.000	**35000.000**
16	15174.798	0.000	0.000	4749.580	0.000	0.000	0.000	315.190	0.000	**20239.569**
17	15174.798	0.000	0.000	5050.573	0.000	0.000	0.000	0.000	0.000	**20225.371**

Stream no.	Water	Hydrogen	Hydrogen peroxide	Sodium Carbonate	Oxygen	HCL	CO2	NaCl	Sodium Percarbonate	Total
18	14719.599	0.000	101.473	5262.020	0.000	0.000	0.000	0.000	0.000	**20083.091**
19	384.600	0.000	0.000	0.000	0.000	207.092	0.000	0.000	0.000	**591.692**
20	384.600	0.000	0.000	0.000	0.000	207.092	0.000	0.000	0.000	**591.692**
21	0.000	0.000	0.000	0.000	0.000	0.000	125.166	0.000	0.000	**125.166**
22	15174.798	0.000	101.473	4960.484	0.000	0.000	125.166	315.190	0.000	**20677.111**
23	15174.798	0.000	101.473	4960.484	0.000	0.000	0.000	315.190	0.000	**20551.945**
24	0.000	0.000	0.000	0.000	0.000	0.000	125.166	0.000	0.000	**125.166**
25	15174.798	0.000	101.473	411.263	0.000	0.000	0.000	5078.997	0.000	**20766.531**
26	15174.798	0.000	101.473	411.263	0.000	0.000	0.000	5078.997	0.000	**20766.531**
27	15174.798	0.000	0.000	200.359	0.000	0.000	0.000	5078.997	0.000	**20454.154**
28	0.000	0.000	0.000	0.000	0.000	0.000	0.000	0.000	312.377	**312.377**
29	50174.798	0.000	0.000	5050.573	0.000	0.000	0.000	0.000	0.000	**55225.371**
30	47184.598	0.000	0.000	4749.580	0.000	0.000	0.000	0.000	0.000	**51934.179**
31	2990.200	0.000	0.000	300.992	0.000	0.000	0.000	0.000	0.000	**3291.192**
32	2990.200	0.000	0.000	0.000	0.000	0.000	0.000	315.190	0.000	**3305.390**

Table S2: Materials and Energy Prices

	Price
**Desalinated Water**	7 €/ton
**Sodium Carbonate**	250 €/ton
**HCl (35%)**	150 €/ton
**Hydrogen**	5000 €/ton
**Sodium Percarbonate**	780 €/ton
**Electricity**	60 €/MWh
**LP Steam**	5.4 €/ton

Table S3: Operational Expenses Breakdown (detailed)

Category	M€/yr	Percentage (%)
**Direct Production Costs**	**6.10**	**26.99**
Fixed costs	0.05	0.24
Patents	0.05	0.24
Variable costs	6.05	26.75
Utilities	1.36	5.99
Maintenace & Repairs	1.13	5.02
Raw Materials	1.75	7.74
Operating Labour	1.81	8.00
**Capital Charges**	**11.42**	**52.89**
**Plant Overhead**	**0.68**	**3.14**
**General Expenses**	**3.39**	**15.71**
Fixed (Sales & Marketing, Engineering, R&D)	1.36	6.28
Variable (Distribution, Training, Management)	2.03	9.42
**Total Manufacturing Costs (TMC)**	**21.59**	**100**

Table S4: Operational Expenses per Unit

**Electrolyser 2MW**						
Electricity	2	MWh/hr	120	€/hr	960000	€/yr
Make Up Water	268.71	kg/hr	1.88	€/hr	15048	€/yr
Sodium Carbonate	211.98	kg/hr	52.99	€/hr	423960	€/yr
Cathode Material (1 year)					2790	€/yr
Total					1401798	€/yr
**Water Evaporator**						
Electricity	25	kWh/hr	1.5	€/hr	12000	€/yr
LP Steam	6.23	ton/hr	33.68	€/hr	269473	€/yr
Total					281473	€/yr
**Acidifier (Mixer)**						
Estimate			100% CAPEX		54011	€/yr
HCl	591	kg/hr	88.65	€/hr	709200	€/yr
Total					763211	€/yr
**IEX**						
Resin (1 per year)	60846.5	kg/yr	837	€/ton	50928	€/yr
**Filter**						
Filter (1 per year)					140000	€/yr
**Dryer**						
Centrifuge Power	30	kW	1.8	€/hr	14400	€/yr
Dryer Power	14.8	kW	0.89	€/hr	7104	€/yr
Total					21504	€/yr
**Pumps**						
Pump1 Power	1.91	kW	0.115	€/hr	917	€/yr
Pump2 Power	1.91	kW	0.115	€/hr	917	€/yr
Pump3 Power	0.02	kW	0.00	€/hr	10	€/yr
Total					1844	€/yr
**Coolers**						
Cooling Water 1	3.25	MW	2.31	€/hr	18481	€/yr
Cooling Water 2	0.566	MW	0.40	€/hr	3214	€/yr
Total					21695	€/yr
**Compressor**						
Power	30	kW	1.8	€/hr	1400	€/yr

Table S5: Equipment List and Cost

Equipment	No. of Units	Cost (M€)	Total Cost (M€)
Electrolyser (2MW)	1	14.636	14.636
Water Evaporator	1	2.633	2.633
Acidifier (Mixer)	1	0.054	0.054
Ion-Exchange (IEX)	2	0.199	0.397
Crystallizer	1	0.384	0.384
Flash 1	1	0.097	0.097
Flash 2	1	0.085	0.085
Filter	1	0.140	0.140
Filtration and Drier	1	0.222	0.222
Pump1	1	0.014	0.014
Pump 2	1	0.014	0.014
Pump 3	1	0.088	0.088
Cooler 1	1	0.279	0.279
Cooler 2	1	0.099	0.099
Degasifier	1	0.168	0.168
Blower	1	0.031	0.031
Donnan Dialysis	1	2.177	2.177
Compressor	1	0.072	0.072
Electrolyte Tank 1	1	0.046	0.046
Electrolyte Tank 2	1	0.046	0.046
**Total Equipment Cost**		**21.683**	
